# Reviews

**Published:** 1880-12

**Authors:** 


### Reviews.

**What to do First in Accidents or Poisoning.** By CHARLES W. DULLES, M. D. Philadelphia: Presley Blakiston, 1012 Walnut street. 1880.

The title of this little book, of half a hundred pages, sufficiently explains its objects and purposes. It is apparently not intended for the physician or surgeon, but rather for the benefit of the laity. It would undoubtedly be well if the information contained within its pages were generally diffused.

---

**Photographic Illustrations of Cutaneous Syphilis.** By GEORGE HENRY FOX, A. M., M. D., Clinical Lecturer on Diseases of the Skin, College Physicians and Surgeons, New York. Forty-eight plates from life, colored by hand. Complete in twelve numbers Price, $2.00 each. New York: E. B. Treat, 757 Broadway.

The previous work by the same author, issued in similar style to the present, has been received with great favor by the profession, and in the further effort to illustrate the syphiloderma, we bespeak a similar reception to the efforts of this laborious dermatologist. We have received Nos. 1, 2 and 3 of the present work. No. 1 gives clear and beautiful plates, illustrating syphiloderma, erythematosum (breast), syph. erythematosum (back), pigmentatio post syphiloderma, leucoderma post syphiloderma, syphiloderma erythematosum; No. 2, plates illustrating syphiloderma papulosum lenticulare, syphiloderma papulosum miliare, syphiloderma papulosum squamosum (breast), syphiloderma papulosum squamosum (shoulder), syphiloderma papulosum; No. 3, plates illustrating syphiloderma papulosum circinatum, syphiloderma papulo-squamosum, syphiloderma papulo-pustulosum, syphiloderma pustulosum. The text accompanying each plate gives a clear exposition of syphilis in its primary and constitutional varieties, and also the latest and best treatment to overcome it. We are convinced, after a careful examination and review of the previous as well as the present work, that the author has a just claim upon the profession for labors which will be sure to assist in the treatment of a most important class of diseases. Vague and imperfect ideas of dermatology are prevalent. The method here adopted will successfully clear up the indefiniteness existing in many minds. The general practitioner as well as the specialist, should possess this valuable contribution to the literature of skin diseases.

**Diseases of the Throat and Nose, including the Pharynx, Larynx, Trachea, Œsophagus, Nasal Cavities and Neck.** By MORELL MACKENZIE, M. D, London, Senior Physician to the Hospital for Diseases of the Throat and Chest, &c (In two volumes.) Vol. 1, Diseases of the Pharynx, Larynx and Trachea. Philadelphia: Presley Blakiston, 1012 Walnut st. 1880.

The present volume is devoted to diseases of the pharynx, larynx and trachea, and is soon to be followed by volume second, which will take up the diseases of the œsophagus, nasal cavities and neck. The wide reputation of the accomplished author is an ample guaranty that the work will constitute the ablest and most exhaustive exposition yet published of the important class of diseases to which it is devoted. The work is based partly on courses of lectures delivered at the London Hospital Medical College, and partly on the Jacksonian Prize Essay on Diseases of the Larynx, awarded by the Royal College of Surgeons, of England. Only one work published in this country, that of Dr. Cohen on Diseases of the Throat, compares favorably in the extent of scientific research and practical experience, with the work of Dr. Mackenzie. The fact that two extensive works on the same class of diseases have been demanded by the profession, shows the amount of study and research directed to this specialty. We commend Dr. Mackenzie's work to the profession. It includes the vast experience of its author, and at this time, when diphtheria, laryngeal croup and like diseases are so prevalent, the practitioner should not fail to avail himself of the best authorities extant to secure information in order to treat successfully diseases which are becoming of such frequent occurrence.

---

**A Practical Treatise on Fractures and Dislocations.** By FRANK H. HAMILTON, M. D. Sixth American edition, revised and improved. Illustrated with three hundred and fifty-two wood cuts. Philadelphia: Henry C. Lea's Sons & Co, 1880.

It is scarcely necessary to do anything more than simply announce that the Sixth Edition of "Hamilton on Fractures and Dislocations," has appeared. The book is known to be the only complete treatise in the English language, or in any language, and needs no recommendation. If there should still be a surgeon who does not have the book in his library, we advise him to get it immediately. A chapter on General Prognosis has been added, other chapters have been rewritten, and the whole book revised. Some new illustrations have been added and others omitted.

---

**Walsh's Physician's Handy Ledger, and Physician's Combined Call Book and Tablet.** Washington, D. C.: RALPH WALSH, M. D., 332 C Street.

These two books are invaluable to the busy practitioner. The call book is one of the best diaries, and its arrangement is by many physicians preferred to any other. If used with the ledger, it wonderfully simplifies the keeping of accounts, and at the same time ensures accuracy.

---

**The Medical Record Visiting List; or Physician's Diary, for 1881.** New York: Wm. Wood & Co.

This new visiting list is the most handsome of all the many diaries offered for physicians' use. It seems to contain all that is necessary in a pocket memorandum of professional visits, and will undoubtedly become a favorite.

---

**Lindsay & Blakiston's Physician's Visiting List for 1881.**

For thirty years this list has held its own against all competitors, and is to-day undoubtedly the most popular visiting list extant.

---

**School and Industrial Hygiene.** By D. F. LINCOLN, M. D., Chairman Department of Health, Social Science Association.

**The Skin in Health and Disease.** By L. DUNCAN BULKLEY, M. D., Attending Physician for Skin and Venereal Diseases at the New York Hospital. Philadelphia: Presley Blakiston, 1012 Walnut street. 1880.

These little works, containing elementary principles on the special subjects of which they treat, constitute Nos. 10 and 12 of the American Health Primers. They are really valuable works, which the profession can consistently recommend to such of their patients as have neither the time nor the means for large and more exhaustive treatises. We recommend them as thoroughly scientific and practical.

---

**A Treatise on the Diseases of the Eye.** By J. SOELBERG WELLS, F.R.C S., with copious additions by CHARLES STEDMAN BULL, A. M., M. D. Henry C. Lea's Son & Co.

Ever since the first edition of this work was issued, it has been regarded as one of the most complete text books on the subject in the English language. Translations from one or two German authorities were perhaps more voluminous, but few writers presented the leading facts of ophthalmology in such a clear and concise manner as did the author. Those who have been students at the Royal Ophthalmic Hospital, recognize the direct and impressive style which characterized also the lectures of this lamented teacher, and doubly regret that his sudden death prevented a revision of his excellent work. But so rapid has been the advance in this department, during the last ten years, that an edition was demanded which presented the more recent phases of this subject. Such a one has lately been given to the English profession, and now Dr. Bull, of New York, offers the present work to American readers. Comparatively few changes have been made in the text, but the editor introduces his comments in such a free but judicious manner as to freshen the parts that might appear antiquated, and thus present to the student a clear and attractive exposition of ophthalmology as it is to-day. The limited space allotted to reviews in the ordinary medical journal, necessitates general statements only, of praise or blame, and it is therefore impossible to mention here in detail, the special advantages of this work or the improvements made in the present edition. We do not hesitate to say, however, that it must be generally regarded as one of the most thorough treatises upon diseases of the eye available to English readers.

